# Peer Review of “The Influence of COVID-19 Vaccination on Daily Cases, Hospitalization, and Death Rate in Tennessee, United States: Case Study”

**DOI:** 10.2196/32461

**Published:** 2021-08-12

**Keywords:** COVID-19, pandemic, vaccination, vaccine, strategy, vaccination strategy, hospitalization, mortality rates, older adults, mortality

*This is a peer-review report submitted for the paper “The Influence of COVID-19 Vaccination on Daily Cases, Hospitalization, and Death Rate in Tennessee, United States: Case Study.”*

## Round 1 Review

### General Comments

This paper [1] addresses an important subject pertaining to vaccination and COVID-19, which has been a major public health concern across the globe for the past year. However, I have a few concerns and comments about the paper concerning the content of the Introduction, Methods, and Results sections as well as the Discussion.

### Specific Comments

#### Major Comments

1. The title should be revised to reflect the analysis performed by the author. The author should consider changing the title to “COVID-19 Vaccination and the Daily Cases, Hospitalizations, and Death Rates: A Case Study of Tennessee in the United States.”
2. I suggest the author provide a brief overview of the COVID-19 pandemic globally and locally in the Introduction.
3. I suggest the author provide a brief description of the study data and how the variables were derived and measured in the Methods section.
4. The author should provide the analytical procedure of the study by describing the statistical methods deployed in the analysis together with the statistical software used. The author did not state whether the analysis is descriptive or inferential and the level of analysis being performed.
5. In the Results section, the author did not provide results for 2020 prior to the onset of vaccination but compared some of the results with December 2020. This will help uncover any changes during the vaccination period.

#### Minor Comments

1. The author should take a critical look at the write-up and provide a thorough proofreading of the paper to correct the several typos and omissions in the text in order to improve clarity and understandability.
2. The vaccination onset in the Abstract section should be 2020, not 2021.
3. The author should change the “mapping” mentioned in the Discussion section to “charts.”
4. Based on the analysis, the author should be careful with the use of “significantly influence” and “impact” throughout the paper.

## Round 2 Review

### General Comments

The author has effectively addressed all my concerns about this paper. The paper should be accepted for publication.
